# The IL-2/IL-2-Receptor Complex in the Maturation of Rat T-Cell Progenitors

**DOI:** 10.1155/1998/95861

**Published:** 1998

**Authors:** Alberto Varas, Teresa Romo, Eva Jiménez, Luis Alonso, Angeles Vicente, Agustín G. Zapata

**Affiliations:** Department of Cell Biology Faculty of Biology Complutense University Madrid 28040 Spain

**Keywords:** Interleukin-2 (IL-2), interleukin-2 receptor (IL-2R), rat, thymocytes

## Abstract

On the basis of both the interleukin-2-receptor (IL-2R) *α*-chain expression on 16-day-old fetal rat thymocytes and the occurrence of interleukin-2 (IL-2) mRNA-containing cells early during rat thymus ontogeny, we have investigated the possible role of IL-2/IL-2R complex in rat T-cell maturation. For this purpose, we analyzed the effects of the addition of either recombinant rat IL- 2 or anti-CD25 (OX-39)-blocking monoclonal antibodies to fetal thymus organ cultures (FTOC), established from 16-day-old rat embryos. IL-2 stimulated the growth of thymocytes and, as a result, induced T-cell differentiation, whereas OX-39 mAb blocked the maturation of thymic-cell progenitors. Accordingly, these results support the involvement of IL-2/IL-2R complex in rat Tcell development.


*Developmental Immunology*, 1998, Vol. 6, pp. 141–147
Reprints available directly from the publisher
Photocopying permitted by license only

© 1998 OPA (Overseas Publishers Association)
N.V. Published by license under the
Harwood Academic Publishers imprint,
part of the Gordon and Breach Publishing Group
Printed in Malaysia

### The IL-2/IL-2-Receptor Complex in the Maturation of Rat T-Cell Progenitors

ALBERTO VARAS, TERESA ROMO, EVA JIMÉNEZ, LUIS ALONSO, ANGELES VICENTE and AGUSTÍN G. ZAPATA*

*Department of Cell Biology, Faculty of Biology, Complutense University, 28040 Madrid, Spain*

*(Received 20 May 1997; In final form 30 May 1997)*

On the basis of both the interleukin-2-receptor (IL-2R) $\alpha$-chain expression on 16-day-old fetal rat thymocytes and the occurrence of interleukin-2 (IL-2) mRNA-containing cells early during rat thymus ontogeny, we have investigated the possible role of IL-2/IL-2R complex in rat T-cell maturation. For this purpose, we analyzed the effects of the addition of either recombinant rat IL-2 or anti-CD25 (OX-39)-blocking monoclonal antibodies to fetal thymus organ cultures (FTOC), established from 16-day-old rat embryos. IL-2 stimulated the growth of thymocytes and, as a result, induced T-cell differentiation, whereas OX-39 mAb blocked the maturation of thymic-cell progenitors. Accordingly, these results support the involvement of IL-2/IL-2R complex in rat T-cell development.

*Keywords:* Interleukin-2 (IL-2), interleukin-2 receptor (IL-2R), rat, thymocytes

### INTRODUCTION

It is now accepted that interleukin-2 (IL-2) is a potent growth factor for mature T cells. However, much controversy has arisen concerning its role, if any, in the first stages of T-cell development. The expression of individual chains ($\alpha$, $\beta$, and $\gamma$) of the IL-2 receptor (IL-2R) on immature thymocytes (Ceredig et al., 1985; Toribio et al., 1989; Kondo et al., 1994; Reya et al., 1996), as well as the ability of these cell subsets to produce IL-2 (Tentori et al., 1988b; Zlotnik et al., 1992) and proliferate in its presence (Ceredig et al., 1989; Toribio et al., 1989; Brooks et al., 1993), support the idea that IL-2 may drive the proliferation and the differentiation of T-cell precursors. In fact, the culture of T-cell progenitors with IL-2 promotes their differentiation to TcR$\alpha\beta$, TcR$\gamma\delta$, and NK cells (Toribio et al., 1988; De la Hera et al., 1989; Brooks et al., 1993; He and Kabelitz, 1995), and *in vivo* or *in vitro* treatments that alter the IL-2/IL-2R complex profoundly modify the T-cell maturation (Jenkinson et al., 1987; Skinner et al., 1987; Tentori et al., 1988a; Plum et al., 1990; Waanders and Boyd, 1990; Zuñiga-Pflücker and Kruisbeek, 1990; Zuñiga-Pflücker et al., 1990; Kroemer et al., 1991; Maslinski et al., 1992). Furthermore, two waves of IL-2 mRNA production,

*Corresponding author.

which correlate well with the differentiation of two waves of T-cell precursors (Jotereau et al., 1987; Penit and Vasseur, 1989), have been reported during fetal thymus development (Montgomery and Dallman 1991; Deman et al., 1994). Nevertheless, gene-disruption experiments suggest that IL-2/IL-2R complex is not required for the generation of normal cell populations in the murine thymus (Schorle et al., 1991; Suzuki et al., 1995; Willerford et al., 1995). In addition, some authors reported the lack of CD25 expression on rat immature thymocytes (Takacs et al., 1988; Kampinga and Aspinall, 1990). However, we conclusively demonstrated the expression of IL-2R$\alpha$ chain on CD4$^-$CD8$^-$CD3$^-$ triple-negative (TN) cells during rat thymus ontogeny. From this basis, we have analyzed the effects of the addition of recombinant rat IL-2 and the blockade of IL-2R by anti-CD25 antibodies on rat thymocyte maturation, using fetal thymus organ cultures (FTOC) established from fetal day-16 thymic lobes.

### RESULTS

### Expression of IL-2 Receptors and IL-2 mRNA on Rat Fetal Thymocytes

The flow cytometrical analysis of rat fetal thymocytes demonstrated that the highest proportion of IL-2R$\alpha$-expressing thymocytes (20-30%) occurred at day 16 of gestation, when most thymocytes (~90%) corresponded to CD3$^-$CD4$^-$CD8$^-$ cells (Figure 1a). At the same stage, 20-25% of total thymic cells contained IL-2 mRNA, as detected by *in situ* hybridization (Figure 1b).

### Thymocyte Development in Rat FTOC

According to the acquisition of CD4, CD8, and TcR$\alpha\beta$ cell markers, rat FTOC mimicked the *in vivo* T-cell development during rat thymus ontogeny. In the first 4 days of culture, there was a gradual decrease of the frequency of CD4$^-$CD8$^-$ double-negative (DN) cells, in correlation with the appearance of immature CD4$^-$CD8$^+$, CD4$^+$CD8$^+$ double-positive (DP), and mature CD4$^+$CD8$^-$ and CD4$^-$CD8$^+$ single-positive (SP) thymocytes (Figure

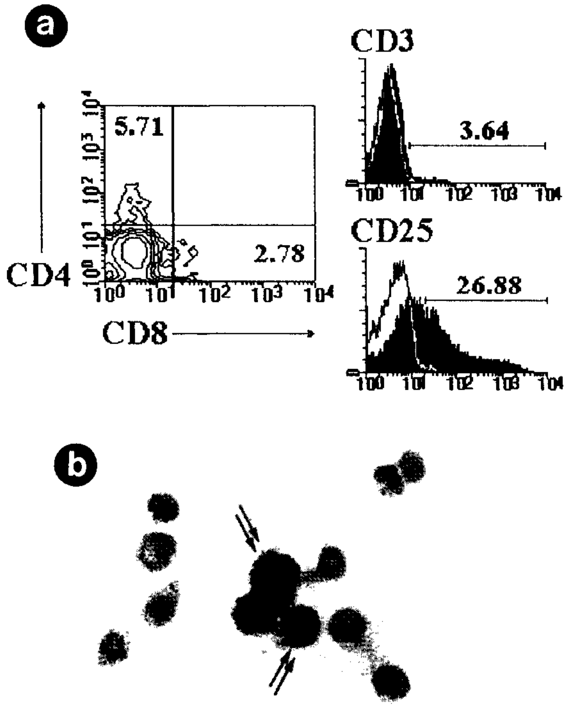

FIGURE 1 (a) Expression of CD4, CD8, CD3, and CD25 antigens on fetal thymocytes from 16-day-old rat embryos. (b) IL-2 mRNA-containing cells (arrows) in thymocyte cytospin preparations from 16-day-old fetal rats.

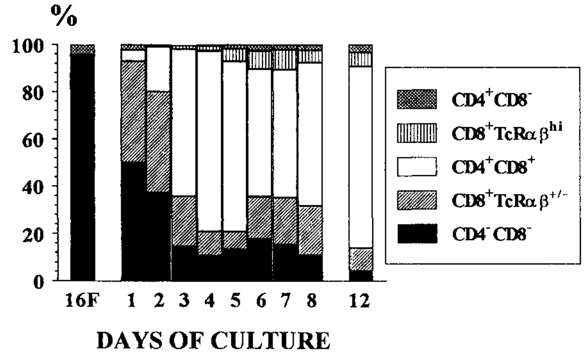

FIGURE 2 Evolution of control rat FTOC. Percentages of the different thymic-cell subsets defined according to the coordinate expression of CD4 and CD8. In these analyses, the subset CD4$^-$CD8$^+$ was subdivided into a CD8$^+$TcR$\alpha\beta^{high}$ cell subpopulation, obtained from gates in double stainings of CD8/TcR$\alpha\beta$, and a CD8$^+$TcR$\alpha\beta^{+/-}$ cell subset, corresponding to immature CD8$^+$ cells and CD8$^+$TcR$\gamma\delta$ thymocytes.

TABLE I CD25 Expression on $CD3^-$ Thymocytes in Rat Fetal Thymus Organ Cultures

| Day 1 | Day 3 | Day 4 | Day 5 | Day 6 | Day 7 | Day 8 | Day 12 |
|---|---|---|---|---|---|---|---|
| 6.72 ± 2.03 | 5.40 ± 0.67 | 3.92 ± 0.60 | 7.43 ± 1.33 | 5.55 ± 0.71 | 3.92 ± 0.53 | 4.58 ± 0.67 | 6.66 ± 0.60 |

*Note*: At different times of culture, thymocytes were stained with anti-CD25 and anti-CD3 antibodies, and the expression of CD25 was analyzed in the $CD3^-$ cell compartment. Data are expressed as the mean ±SEM from five to seven independent experiments.

2). As occurs *in vivo* (Vicente et al., manuscript submitted), in those days of culture (5 to 7) equivalent to the perinatal period, a new signal of expansion occurred in rat FTOC, inducing a transient increase of the DN-cell subset and its differentiation to immature $CD8^+$, DP, and mature SP thymocytes in the following days (Figure 2). At that time, the percentage of $CD25^+$, which had gradually diminished during the first 4 days of culture, sharply increased also in the $CD3^-$ cell compartment (Table I).

### Effects of IL-2 on the Development of Thymic Major Cell Subpopulations

The number of cells per thymic lobe increased after 1 day of treatment with IL-2, remaining unchanged at day 3 and even decreasing after 5 to 7 days of culture. However, one more week of culture in the presence of IL-2 increased again the thymic cellularity (Figure 3).

One day of culture with IL-2 basically induced an increase in the numbers of DN and $CD4^+$ cells (Figure 3). At day 3, the absolute numbers of the different thymocyte subsets hardly changed, excepting for the slight increase in mature $CD8^+$ thymocytes. This cell subset remained unaltered after 5 days of treatment, whereas the cell numbers of the rest of thymocyte subpopulations decreased (Figure 3). By day 7 of culture, the number of DN cells reached control values and that of mature $CD8^+$ thymocytes increased. Five more days of treatment with IL-2 induced a cell expansion affecting to all thymocyte subsets, but in a higher proportion to the DN-cell subpopulation (Figure 3).

### Effects of Anti-CD25 Treatment on Thymocyte Maturation

Anti-CD25 treatment was carried out by adding OX-39 mAb, known by blocking the binding of IL-2 to

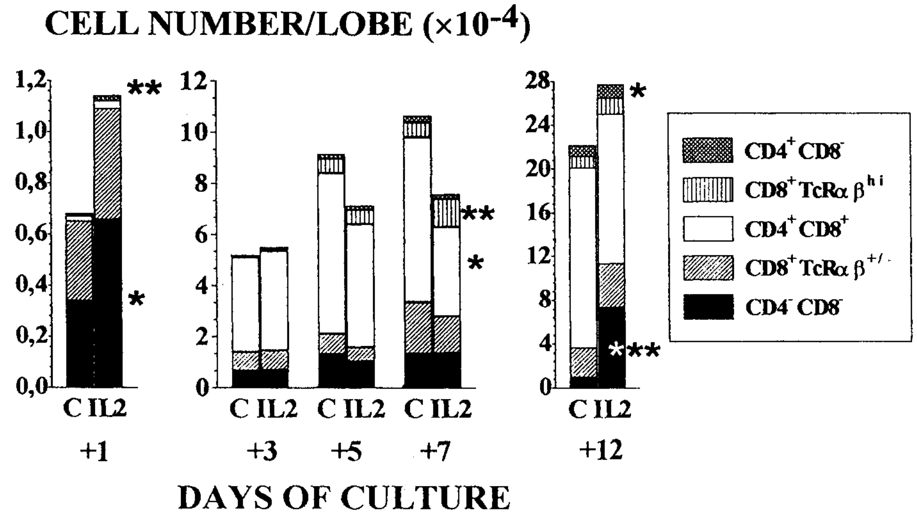

FIGURE 3 Absolute numbers of different thymic-cell subpopulations in control (left bar) and IL-2-treated (right bar) FTOC for 1, 3, 5, 7, and 12 days. Thymocyte subsets were defined in double stainings by expresion of CD4, CD8 and TcR$\alpha\beta$. At each time point, the data represented are the mean of three to five independent experiments. * $p \leq 0.05$; ** $p \leq 0.01$; *** $p \leq 0.001$ (Student's $t$ test).

TABLE II Cell Number of Thymocyte Subsets after Anti-CD25 Treatment

| Time | | Phenotype CD4$^-$CD8$^-$ | CD8$^+$TcR$\alpha\beta^{+/-}$ | CD4$^+$CD8$^+$ | CD8$^+$TcR$\alpha\beta^{high}$ | CD4$^+$CD8$^-$ | Total |
|---|---|---|---|---|---|---|---|
| Day 1 | Control | 0.58 ± 0.11 | 0.50 ± 0.09 | 0.06 ± 0.01 | — | 0.03 ± 0.01 | 1.17 ± 0.22 |
| | OX-39 | 0.35 ± 0.07 | 0.21 ± 0.04[b] | 0.02 ± 0.01[a] | — | 0.02 ± 0.00 | 0.60 ± 0.12[a] |
| Day 3 | Control | 0.63 ± 0.06 | 1.03 ± 0.10 | 2.94 ± 0.38 | 0.08 ± 0.01 | 0.01 ± 0.00 | 4.69 ± 0.55 |
| | OX-39 | 0.61 ± 0.06 | 0.76 ± 0.12[a] | 1.18 ± 0.32[b] | 0.06 ± 0.01 | 0.01 ± 0.00 | 2.62 ± 0.52[b] |
| Day 5 | Control | 1.10 ± 0.30 | 0.66 ± 0.22 | 6.46 ± 1.43 | 0.43 ± 0.07 | 0.13 ± 0.06 | 8.78 ± 2.10 |
| | OX-39 | 0.63 ± 0.26 | 0.14 ± 0.02[b] | 2.74 ± 0.86[b] | 0.08 ± 0.02[c] | 0.02 ± 0.01[a] | 3.61 ± 1.12[b] |
| Day 7 | Control | 1.20 ± 0.18 | 1.92 ± 0.30 | 4.41 ± 1.22 | 0.93 ± 0.20 | 0.30 ± 0.12 | 8.76 ± 1.73 |
| | OX-39 | 0.88 ± 0.19 | 0.42 ± 0.11[b] | 0.62 ± 0.16[b] | 0.15 ± 0.03[c] | 0.06 ± 0.01[b] | 2.13 ± 0.41[c] |
| Day 12 | Control | 0.76 ± 0.16 | 2.22 ± 0.44 | 20.05 ± 4.64 | 1.90 ± 0.52 | 1.01 ± 0.30 | 25.94 ± 6.08 |
| | OX-39 | 0.46 ± 0.15 | 0.21 ± 0.07[c] | 0.62 ± 0.25[c] | 0.08 ± 0.03[c] | 0.03 ± 0.02[c] | 1.40 ± 0.55[c] |

*Note*: Values represent the mean cell number ($\times 10^{-4}$) per lobe ±SEM from three to six independent experiments.
[a]$p \leq 0.05$;
[b]$p \leq 0.01$;
[c]$p \leq 0.001$ (Student's *t* test).

high-affinity IL-2 receptors (Paterson et al., 1987; Somoza et al., 1990).

From the beginning of culture, the addition of OX-39 mAb markedly inhibited viable cell yield in rat FTOC. The reduction in thymic cellularity was increasing to reach the highest effect after 12 days of treatment (Table II).

In the continuous presence of OX-39, the absolute numbers of all thymocyte subsets were always lower than in control FTOC, being, at any time, the DN-cell subpopulation the least affected by the treatment (Table II). However, whereas the differentiation of the second wave of T-cell progenitors was totally inhibited, the cell precursors present in 16-day-old fetal thymus partially matured, presumably because some of them had already overcome the CD25$^+$ stage (Table II).

### DISCUSSION

The current data indicate that rat T-cell precursors (TN cells) express IL-2 receptors, a fact previously denied by other authors (Takacs et al., 1988; Kampinga and Aspinall, 1990), although repeatedly reported in mice, chickens, and humans (Ceredig et al., 1985; Toribio et al., 1989; Zuñiga-Pflücker et al., 1990; Fedecka-Brunner et al., 1991). In agreement with our results, Brocke et al. (1987) found IL-2R$\alpha$-bearing cells in the thymus of 16-day-old fetal rats. However, the immunohistological demonstration of CD4 and CD8 expression in these thymic lobes was misinterpreted, concluding that CD25$^+$ thymocytes corresponded to mature thymocytes. Obviously, as our results demonstrate, the rat thymic primordium does not contain mature SP thymocytes but DN cells, some of which, in progression to the DP-cell subset, could already express CD4 and/or CD8 molecules in their cytoplasm.

On the other hand, since IL-2 supports the growth of rat thymocytes in FTOC, in a way that can be blocked by anti-CD25 antibodies, IL-2 receptors expressed on rat fetal thymocytes seem to be functional. Contradictory results have been reported on the ability of IL-2 to induce proliferative responses in early thymocytes (Raulet, 1985; von Boehmer et al., 1985; de la Hera et al., 1989; Toribio et al., 1989; Brooks et al., 1993). Presumably, as recognized by many authors, this induction is dependent on thymic stromal cells or an intact thymic microenvironment, as provided by organ cultures (De la Hera et al., 1989; Ceredig et al., 1989; Zuñiga-Pflücker et al., 1990). However, the continuous addition of IL-2 to rat FTOC inhibits thymocyte growth and T-cell maturation, as also reported in mouse organ cultures (Skinner et al., 1987; Plum et al., 1990; Waanders and Boyd, 1990). The generation of LAK cells has been pointed out to be involved in the depletion of thymocytes and the subsequent inhibition of T-cell differentiation (Skinner et al., 1987). Alternatively, negative signals transduced via the IL-2R in response to the IL-2 concentrations used in these experiments, which are not continuously present *in vivo*, could also explain these results (Waanders and Boyd, 1990). In fact, the inhibition of cell proliferation (Suwa et al., 1995) and the induction of apoptosis (Lenardo, 1991; Migliorati et al., 1993) have been reported after IL-2 treatment.

Our results obtained after IL-2 and anti-CD25 treatments, in agreement with previous findings in both humans and mice (Toribio et al., 1988; Zuñiga-Pflücker and Kruisbeek, 1990; Wilson et al., 1994) also indicate that IL-2 promotes the differentiation of thymic-cell precursors, as a consequence of its capacity to stimulate cell proliferation.

Taken together, these results support a role for the IL-2/IL-2R complex in the intrathymic maturation of rat T-cell precursors.

### MATERIALS AND METHODS

### Animals

Wistar rats were maintained in our animal facilities. Fetuses at day 16 of gestation were obtained from timed pregnancies. The day of finding a vaginal plug was designated day 0 of gestation.

### Fetal Thymus Organ Cultures

Thymic lobes were aseptically removed from 16-day-old rat embryos, trimmed of surrounding mechenchyme, and organ-cultured as follows. Four to six thymic lobes were placed on the surface of polycarbonate filters (Millipore Ibérica, Spain) supported by stainless steel screen pieces. Lobes were cultured in the central well of organ tissue culture dishes (Becton-Dickinson, Spain) with 1 ml of RPMI 1640 medium (2 mM L-glutamine) supplemented with sodium pyruvate (1 mM), streptomycin (100 $\mu$g/ml), penicillin (100 U/ml) (all reagents: Gibco BRL, France), and 10% FCS (Biosys, France). The cultures were grown in a humidified incubator in 10% $CO_2$ in air at 37°C, and the medium was replaced daily. The control cultures were done as described, the IL-2-treated organ cultures were performed at a concentration of 20 U/ml of recombinant rat IL-2 (Serotec, UK), and the OX-39-treated cultures were carried out in complete medium supplemented with 50% culture supernatant from OX-39 hybridoma (anti-rat CD25). In this case, control cultures made in parallel were supplemented with 50% culture supernatant from an irrelevant hybridoma (OX-14, anti-rat IgG2b).

### Cell-Surface Staining

At various times through the culture period, a cell suspension was made of thymic lobes, total cell count was done, and the expression of CD4 (OX-38-PE), CD8 (OX-8-FITC), TcR$\alpha\beta$ (R. 73-PE), CD3 (G4.18-FITC), and CD25 (OX-39-PE) (all from Pharmingen, USA) were analyzed. Flow cytometric analysis was carried out on a FACScan (Becton-Dickinson, USA). Dead cells were excluded from data acquisition on the basis of forward/side scatter and propidium iodide staining.

### *In Situ* Hybridization

Fetal thymocytes from day 16 of gestation were isolated, cytospun onto slides, and fixed in paraformaldehyde (4% in PBS) during 30 min at room temperature. After permeabilization with proteinase K (2 $\mu$g/ml) in Tris-EDTA during 20 min at room temperature, cells were acetylated and dehydrated. Hybridization was carried out at 37°C in 5× SSC, 30% formamide, herring DNA (10 mg/ml), t-RNA (10 mg/ml), and 5 $\mu$g/ml of digoxigenin-labeled cDNA probe for rat IL-2. Slides were washed in 30% formamide in 2× SSC at room temperature and 42°C during 5 and 15 min, respectively. Anti-digoxigenin antibodies conjugated to alkaline phosphatase (Boehringer Mannheim, Germany) were used for the immunological detection according to the commercial supplier's recommendations.

### *References*

Brocke S., Takacs L., Gerdes J., Osawa H., and Diamanstein T. (1987). The ontogeny of the interleukin 2 receptor expression and the interleukin 2 responsiveness in the rat thymus. Immunobiol. **174**: 266-273.

Brooks C. G., Georgiou A., and Jordan R. K. (1993). The majority of immature fetal thymocytes can be induced to proliferate to IL-2 and differentiate into cells indistinguishable from mature natural killer cells. J. Immunol. **151**: 6645-6656.

Ceredig R., Lowenthal J. W., Nabholz M., and MacDonald H. R. (1985). Expression of IL-2 receptors as a differentiation marker on intrathymic stem cells. Nature **314**: 98-100.

Ceredig R., Medveczky J., and Skulimowski A. (1989). Mouse fetal thymus lobes cultured with IL-2 generate CD3+, TcR$\gamma\delta$-expressing CD4−CD8+ and CD4−CD8− cells. J. Immunol. **142**: 3353-3360.

De la Hera A., Marston W., Aranda C., Toribio M. L., and Martínez-A C. (1989). Thymic stroma is required for the development of human T cell lineages in vitro. Int. Immunol. **1**: 471-478.

Deman J., Humblet C., Martin M. T., Boniver J., and Defresne M. P. (1994). Analysis by in situ hybridization of cytokine mRNA expression in the murine developing thymus. Int. Immunol. **6**: 1613-1619.

Fedecka-Brunner B., Penningers J., Vaigot P., Lehmann A., Martínez-A C., and Kroemer G. (1991). Developmental expression of IL-2-receptor light chain (CD25) in the chicken embryos. Devel. Immunol. **1**: 237-242.

He W., and Kabelitz D. (1995). Differential effects of Interleukin-2 and Interleukin-7 on the induction of CD4 and CD8 expression by double-negative human thymocytes. Scand. J. Immunol. **41**: 309-312.

Jenkinson E. J., Kingston R., and Owen J. J. T. (1987). Importance of IL-2 receptors in intra-thymic generation of cells expressing T-cell receptors. Nature **329**: 160-162.

Jotereau F., Henze F., Salomon-Vie V., and Gascan H. (1987). Cell kinetics in the fetal mouse thymus: Precursor cell input, proliferation and emigration. J. Immunol. **138**: 1026-1030.

Kampinga J., and Aspinall R. (1990). Thymocyte differentiation and thymic microenvironment development in the fetal rat thymus: an immunohistological analysis. In Thymus Update, Vol. 3, Kendall M.D., and Ritter A., Eds. London: Harwood Academic Publishers), pp. 149-186.

Kondo M., Ohashi Y., Tada K., Nakamura M., and Sugamura K. (1994). Expression of the mouse interleukin-2 receptor $\gamma$ chain in various cell populations of the thymus and spleen. Eur. J. Immunol. **24**: 2026-2030.

Kroemer G., Cid R., Moreno de Alboran I., Gonzalo J. A., Iglesias A., Martínez-A. C., and Gutierrez-Ramos C. (1991). Immunological self-tolerance: An analysis employing cytokines or cytokine receptors encoded by transgenes or a recombinant vaccinia virus. Immunol. Rev. **122**: 174-204.

Lenardo M. J. (1991). Interleukin 2 programs mouse $\alpha\beta$ T lymphocytes for apoptosis. Nature **353**: 858-861.

Maslinski W., Murphy J. R., and Strom T. B. (1992). Intoxication of high affinity IL-2 receptor positive thymocyte blocks early stages of T cell maturation. Int. Immunol. **4**: 509-517.

Migliorati G., Nicoletti I., Pagliacci M. C., D'adamio L., and Riccardi C. (1993). Interleukin-2 induces apoptosis in mouse thymocytes. Cell. Immunol. **146**: 52-61.

Montgomery R. A., and Dallman M. J. (1991). Analysis of cytokines gene expression during fetal thymic ontogeny using the polimerase chain reaction. J. Immunol. **147**: 554-560.

Paterson D. J., Jeffreis W. A., Green J. R., Brandon M. R., Corthesy P., Puklavec M., and Williams A. F. (1987). Antigens of activated rat T lymphocytes including a molecule of 50,000 Mr detected only on CD4 positive T blasts. Mol. Immunol. **24**: 1281-1290.

Penit C., and Vasseur F. (1989). Cell proliferation and differentiation in the fetal and early postnatal mouse thymus. J. Immunol. **142**: 3369-3377.

Plum J., Koning F., Leclercq G., Tison B., and De Smedt M. (1990). Expansion of large granular lymphocytes in IL-2 driven 14-day-old fetal thymocytes in organ cultures. J. Immunol. **144**: 3710-3717.

Raulet D. H. (1985). Expression and function of IL-2 receptors on immature thymocytes. Nature **314**: 101-103.

Reya T., Yangsnyder J. A., Rothenberg E. V., and Carding S. R. (1996). Regulated expression and function of CD122 (IL-2/IL-15R$\beta$) during lymphoid development. Blood **87**: 190-201.

Schorle H., Holtschke T., Hünig T., Schimpl A., and Horak I. (1991). Development and function of T cells in mice rendered interleukin-2 deficient by gene targeting. Nature **352**: 621-624.

Skinner M., Le Gros G., Marbrook J., and Watson J. D. (1987). Development of fetal thymocytes in organ culture. Effect of interleukin 2. J. Exp. Med. **165**: 1481-1493.

Somoza C., Fernández-Ruiz E., Rebollo A., Sanz E., Ramírez F., and Silva A. (1990). OX-48, a monoclonal antibody against a 70,000 MW rat activation antigen expressed by T cells bearing the high-affinity interleukin-2 receptor. Immunology **70**: 210-215.

Suwa H., Tanaka T., Kitamura F., Shiohara T., Kuida K., and Miyasaka M. (1995). Dysregulated expression of the IL-2 receptor $\beta$-chain abrogates development of NK cells and Thy-1$^+$ dendritic epidermal cells in transgenic mice. Int. Immunol. **7**: 1441-1449.

Suzuki H., Kündig T. M., Furlonger C., Wakeham A., Timms E., Matsuyama T., Schmits R., Simard J. J. L., Ohashi P. S., Griesser H., Taniguchi T., Paige C. J., and Mak T. W. (1995). Deregulated T cell activation and autoimmunity in mice lacking IL-2 receptor $\beta$. Science **268**: 1472-1476.

Takacs L., Ruscetti F. W., Kovacs E. J., Rocha B., Brocke S., Diamanstein T., and Mathieson B. J. (1988). Immature, double negative (CD4$^-$CD8$^-$) rat thymocytes do not express IL-2 receptors. J. Immunol. **141**: 3810-3818.

Tentori L., Longo D. A., Zuñiga-Pflücker J. C., Wing C., and Kruisbeek A. M. (1988a). Essential role of the interleukin 2-interleukin 2 receptor pathway in thymocyte maturation in vivo. J. Exp. Med. **168**: 1741-1747.

Tentori L., Pardoll D. M., Zuñiga-Pflücker J. C., Hu-Li J., Paul W. E., Bluestone J. A., and Kruisbeek A. M. (1988b). Proliferation and production of IL-2 and B cell stimulatory factor/IL-4 in early fetal thymocytes by activation through Thy-1 and CD3. J. Immunol. **140**: 1089-1094.

Toribio M. L., Alonso J. M., Bárcena A., Gutiérrez J. C., Hera de la A., Marcos M. A. R., Marquez C., and Martinez-A. C. (1988). Human T-cell precursors: Involvement of the IL-2 pathway in the generation of mature cells. Immunol. Rev. **104**: 55-79.

Toribio M. L., Gutierrez-Ramos J. C., Pezzi L., Marcos M. A. R., and Martinez-A C. (1989). Interleukin-2-dependent autocrine proliferation in T-cell development. Nature **342**: 82-85.

Von Boehmer H., Crisanti A., Kisielow P., and Haas W. (1985). Absence of growth by most receptor-expressing fetal thymocytes in the presence of interleukin-2. Nature **314**: 539-540.

Waanders G. A., and Boyd R. L. (1990). The effects of interleukin 2 on early and late thymocyte differentiation in foetal thymus organ culture. Int. Immunol. **2**: 461-468.

Willerford D. M., Chen J., Ferry J. A., Davidson L., Ma A., and Alt F. W. (1995). IL-2R$\alpha$ chain regulates the size and content of the peripheral lymphoid compartment. Immunity **3**: 521-530.

Wilson A., Corthesy P., Reichenbach P., Macdonald H. R., and Nabholz M. (1994). Interleukins (IL)-1 and IL-2 control IL-2 receptor $\alpha$ and $\beta$ expression in immature thymocytes. Eur. J. Immunol. **24**: 1729-1735.

Zlotnik A., Godfrey D. I., Fischer M., and Suda T. (1992). Cytokine production by mature and immature $CD4^-CD8^-$ T cells. $\alpha\beta$-T cell receptor $^+CD4^-CD8^-$ T cells produce IL-4. J. Immunol. **149**: 1211-1215.

Zuñiga-Pflücker J. C., and Kruisbeek A. M. (1990). Intrathymic radioresistant stem cells follow an IL-2/Il-2R pathway during thymic regeneration after sublethal irradiation. J. Immunol. **144**: 3736-3740.

Zuñiga-Pflücker J. C., Smith K. A., Tentori L., Pardoll D. M., Longo D., and Kruisbeek A. M. (1990). Are the IL-2 receptors expressed in the murine fetal thymus functional?. Devel. Immunol. **1**: 59-66.

